# Supplementary material for: Modeling glioblastoma heterogeneity as a dynamic network of cell states
Source: Mol Syst Biol. 2021 Sep 16;17(9):e10105. doi: 10.15252/msb.202010105 (PMC8444284; doi:10.15252/msb.202010105)
Supplement: Supplementary file 6 — Source Data for Figure 5 [file MSB-17-e10105-s004.zip › Figure5A_sourcedata/GSEA_3071/hallmarks_stateIII.GseaPreranked.1623742540963/HALLMARK_KRAS_SIGNALING_UP.html]

Details for gene set HALLMARK\_KRAS\_SIGNALING\_UP[GSEA]

|  || Dataset | state33071 |
| Phenotype | NoPhenotypeAvailable |
| Upregulated in class | na\_neg |
| GeneSet | HALLMARK\_KRAS\_SIGNALING\_UP |
| Enrichment Score (ES) | -0.4490341 |
| Normalized Enrichment Score (NES) | -2.4803631 |
| Nominal p-value | 0.0 |
| FDR q-value | 2.0000002E-4 |
| FWER p-Value | 0.001 |
Table: GSEA Results Summary

  

Fig 1: Enrichment plot: HALLMARK\_KRAS\_SIGNALING\_UP      
 Profile of the Running ES Score & Positions of GeneSet Members on the Rank Ordered List

  

| PROBE | GENE SYMBOL | GENE\_TITLE | RANK IN GENE LIST | RANK METRIC SCORE | RUNNING ES | CORE ENRICHMENT || 1 | HBEGF |  |  | 441 | -0.251 | -0.1043 | No |
| 2 | ITGB2 |  |  | 514 | -0.255 | -0.1132 | No |
| 3 | ETV5 |  |  | 818 | -0.270 | -0.1812 | No |
| 4 | KLF4 |  |  | 973 | -0.278 | -0.2104 | No |
| 5 | CDADC1 |  |  | 1019 | -0.280 | -0.2114 | No |
| 6 | PRRX1 |  |  | 1032 | -0.281 | -0.2038 | No |
| 7 | CPE |  |  | 1081 | -0.284 | -0.2054 | No |
| 8 | TFPI |  |  | 1141 | -0.287 | -0.2098 | No |
| 9 | BIRC3 |  |  | 1469 | -0.304 | -0.2826 | No |
| 10 | ETV1 |  |  | 1495 | -0.306 | -0.2775 | No |
| 11 | HKDC1 |  |  | 1523 | -0.307 | -0.2728 | No |
| 12 | CAB39L |  |  | 1559 | -0.310 | -0.2701 | No |
| 13 | SPRY2 |  |  | 1592 | -0.311 | -0.2665 | No |
| 14 | PEG3 |  |  | 1754 | -0.322 | -0.2959 | No |
| 15 | ID2 |  |  | 1780 | -0.323 | -0.2900 | No |
| 16 | ANKH |  |  | 1978 | -0.334 | -0.3282 | No |
| 17 | ITGBL1 |  |  | 2093 | -0.341 | -0.3447 | No |
| 18 | TRIB2 |  |  | 2105 | -0.342 | -0.3346 | No |
| 19 | PLAUR |  |  | 2181 | -0.347 | -0.3407 | No |
| 20 | IL7R |  |  | 2270 | -0.355 | -0.3500 | No |
| 21 | CTSS |  |  | 2434 | -0.368 | -0.3781 | No |
| 22 | F13A1 |  |  | 2535 | -0.376 | -0.3897 | No |
| 23 | DNMBP |  |  | 2549 | -0.377 | -0.3787 | No |
| 24 | SCG5 |  |  | 2597 | -0.382 | -0.3763 | No |
| 25 | PTCD2 |  |  | 2609 | -0.383 | -0.3647 | No |
| 26 | LIF |  |  | 2908 | -0.409 | -0.4261 | No |
| 27 | EMP1 |  |  | 2998 | -0.420 | -0.4331 | Yes |
| 28 | VWA5A |  |  | 3002 | -0.420 | -0.4179 | Yes |
| 29 | CCND2 |  |  | 3037 | -0.425 | -0.4106 | Yes |
| 30 | TNFAIP3 |  |  | 3088 | -0.430 | -0.4072 | Yes |
| 31 | GPRC5B |  |  | 3092 | -0.430 | -0.3916 | Yes |
| 32 | TRIB1 |  |  | 3097 | -0.431 | -0.3763 | Yes |
| 33 | SPON1 |  |  | 3187 | -0.441 | -0.3826 | Yes |
| 34 | BMP2 |  |  | 3307 | -0.462 | -0.3958 | Yes |
| 35 | PCSK1N |  |  | 3346 | -0.470 | -0.3877 | Yes |
| 36 | ADGRA2 |  |  | 3376 | -0.475 | -0.3772 | Yes |
| 37 | PLAU |  |  | 3435 | -0.488 | -0.3736 | Yes |
| 38 | TSPAN7 |  |  | 3528 | -0.520 | -0.3776 | Yes |
| 39 | DCBLD2 |  |  | 3543 | -0.525 | -0.3613 | Yes |
| 40 | SOX9 |  |  | 3560 | -0.531 | -0.3453 | Yes |
| 41 | NRP1 |  |  | 3562 | -0.532 | -0.3254 | Yes |
| 42 | ITGA2 |  |  | 3589 | -0.542 | -0.3115 | Yes |
| 43 | GYPC |  |  | 3598 | -0.546 | -0.2929 | Yes |
| 44 | PLAT |  |  | 3600 | -0.547 | -0.2724 | Yes |
| 45 | RGS16 |  |  | 3616 | -0.555 | -0.2552 | Yes |
| 46 | DUSP6 |  |  | 3648 | -0.577 | -0.2412 | Yes |
| 47 | CFH |  |  | 3653 | -0.582 | -0.2202 | Yes |
| 48 | G0S2 |  |  | 3728 | -0.637 | -0.2151 | Yes |
| 49 | SATB1 |  |  | 3732 | -0.644 | -0.1915 | Yes |
| 50 | AKAP12 |  |  | 3740 | -0.660 | -0.1682 | Yes |
| 51 | MAP3K1 |  |  | 3796 | -0.737 | -0.1545 | Yes |
| 52 | KIF5C |  |  | 3798 | -0.739 | -0.1267 | Yes |
| 53 | INHBA |  |  | 3801 | -0.746 | -0.0989 | Yes |
| 54 | CXCR4 |  |  | 3802 | -0.750 | -0.0705 | Yes |
| 55 | IGFBP3 |  |  | 3903 | -1.199 | -0.0508 | Yes |
| 56 | SPP1 |  |  | 3920 | -1.508 | 0.0023 | Yes |
Table: GSEA details [plain text format]

  

Fig 2: HALLMARK\_KRAS\_SIGNALING\_UP: Random ES distribution      
 Gene set null distribution of ES for **HALLMARK\_KRAS\_SIGNALING\_UP**
